# Copper Beaten Skull! Can It be a Usual Appearance?

**DOI:** 10.5005/jp-journals-10005-1233

**Published:** 2014-04-26

**Authors:** Vela Desai, Smita R Priyadarshini, Rajeev Sharma

**Affiliations:** Professor and Head, Department of Oral Medicine and Radiology, Jaipur Dental College, Jaipur, Rajasthan, India; Senior Lecturer, Department of Oral Medicine and Radiology, Institute of Dental Sciences, Bhubaneswar, Odisha, India; Senior Lecturer, Department of Oral Medicine and Radiology, Jaipur Dental College, Jaipur, Rajasthan, India

**Keywords:** Copper beaten skull, Intracranial tension, Cranio-synostosis

## Abstract

‘Copper beaten’ skull refers to the prominent convolutional markings seen in multiple bones of the skull. Underlying cause is thought to be related to increased intracranial pressure resulting from such processes as craniosynostosis, obstructive hydrocephalus and/or intracranial masses. However, the copper beaten appearance of the skull has poor sensitivity in detecting increased intracranial pressure as such an appearance can also be seen in normal patients. In this article, we have reported a case of a 5 years old child with classical features of beaten silver skull.

**How to cite this article: **Desai V, Priyadarshini SR, Sharma R. Copper Beaten Skull! Can It be a Usual Appearance? Int J Clin Pediatr Dent 2014;7(1):47-49.

## INTRODUCTION

‘Copper beaten’ skull refers to the prominent convolutional markings seen in multiple bones of the skull. Underlying cause is thought to be related to increased intracranial pres­sure resulting from such processes as craniosynostosis, obstructive hydrocephalus and/or intracranial masses.

The skull radiographs can be used as an important diag­nostic method for detecting increased intracranial pressure but it could also be seen as an appearance in normal patients.

## CASE REPORT

A 5-year-old male reported to the department of oral medicine and radiology with the chief complaint of mal-aligned teeth. He was born to nonconsanguineous parents and had a younger sibling.

His appearance was slightly different to children of his age, with protruding eyes. History revealed that the child though normal at birth (2 kg) but on history features started appearing with age. Medical, family or dental history was noncontributory. The personal history revealed good oral hygiene and no pernicious habits, like thumb sucking or tongue thrusting.

**Fig. 1:**
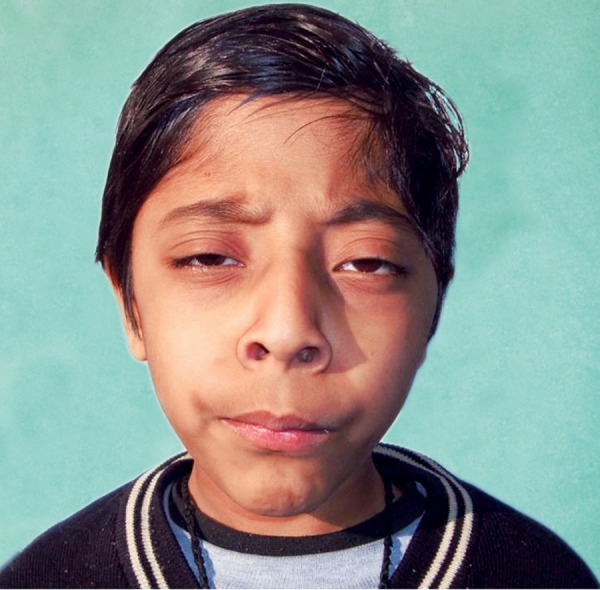
Profile view of the patient

On examination, gross facial asymmetry was seen, with fat nasal bridge, dolichocephalic skull, convex facial Profile and incompetent lips (Fig. 1).

The cranial vault gave the impression of scaphocephaly with signs of synostosis of the sagittal suture. Slight hyper-telorism, proptotic eyes, dystopia of the inner canthus, blep-haroptosis of the upper lid and antimongoloid slant of the palpebral fissures. The auricles were posteriorly positioned.

**Fig. 2:**
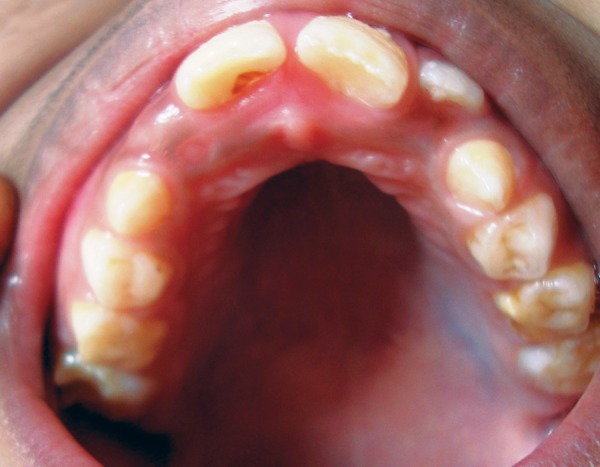
Intraoral examination

**Fig. 3:**
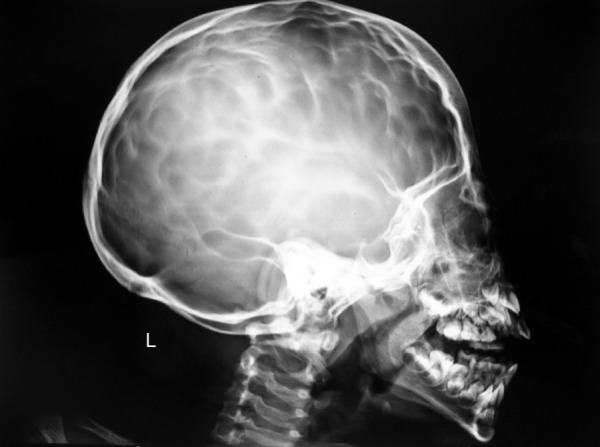
Extraoral radiograph: lateral skull view

Nasal bridge was depressed and nostrils were fared. The mandible was retrognathic, with midfacial hypoplasia with defcient zygomas, lips at rest position presented a fishmouth-like picture, and incompetent lips. The maxillary dental arch was narrow and the palatal vault was very high and narrow. Molars and canines were at class II relationships with anterior open bite (Fig. 2). The patient height was similar to kids of his age. The shoulders were droopy and his developmental milestones were normal.

Patient was advised for lateral cephalograms and AP to evaluate the facial Profile and for any pathology related to the skull. All the investigations were done with due consent of the patient. Anteroposterior (AP) view showed a well corticated small bony fragment at the tip of the 2nd cervical vertebrae suggestive of OS odontium. There was scalloping of the inner table of clavarium along with convolutional markings. Lateral cephalograms showed hypertrophy of the adenoids with slight enlargement of pituitary fossa (Fig. 3).

**Fig. 4:**
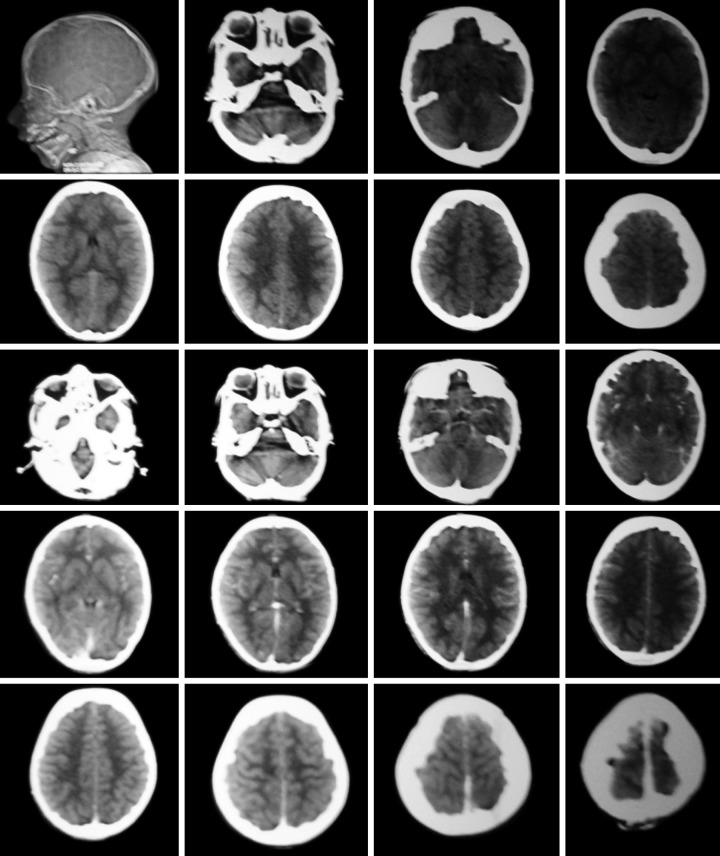
Computed tomography scan

He was referred to the ENT specialist for evaluation but was noncontributory. Computed tomography (CT) scan was suggested to rule out raised intracranial tension which revealed no definitive evidence of intracranial pathology (Fig. 4).

Keeping all this features under consideration the patient was diagnosed for a variation of beaten silver skull. Differen­tial diagnosis for this uncommon presentation consists of craniosynostosis, obstructive hydrocephalus or intracranial tumors. These markings are most prominent during periods of rapid brain growth so this patient is also kept under obser­vation to evaluate any further change in the features or appearance of any abnormality or complications.

## DISCUSSION

There are various skull appearances which can be associated to various syndromes like beaten silver skull in curzons syndrome, tram-line appearance in Sturge-Weber syndrome, calcification of falx cerebri in basal cell nevus syndrome, dolicocephalic skull in Marfans syndrome.

Variations and abnormalities of skull appearance and shape are generally related to a primary maldevelopment of the brain. This is the result of growing brain which exerts pressure on the malleable cranium, producing a pattern known as the copper-beaten skull (also known as beaten brass skull). These convolutional markings may be normal during periods of rapid brain growth between age 2 and 3, and 5 and 7 years.^1^ They become less prominent after approximately 8 years of age. These are usually confined to the posterior part of the skull's inner table but, in this patient, this appearance was generalized over the skull.

Pathology mostly related to increase in this pressure may be related to but are not limited to:

- *Craniosynostosis*: It refers to premature closure of the cranial sutures. The skull shape then undergoes characteristic changes depending on which suture(s) close early. The sagittal suture is most commonly involved (50%), where lateral growth of the skull is arrested while anteroposterior growth continues, producing a narrow elongated skull known as scaphocephaly (meaning boat-shaped) or dolichocephaly. As seen in the present case, the next commonest sutures in terms of involvement are: coronal—20%; lambdoid—5% metopic—5%.
- b. *Obstructive hydrocephalus*: It (also known as non-communicating hydrocephalus) is simply hydrocephalus due to obstruction of CSF fow out of the ventricular system.
- c. *Intracranial masses*: In children <18 months, the presence of a diffuse copper-beaten pattern on skull radiography, together with narrowing of the basal cisterns and obliter­ation of the anterior sulci, increases the likelihood of raised intracranial pressure.^1,2^ Thus, it can be considered as an important differential diagnosis in this case.

Additional findings associated with a chronic increase in intracranial pressure include macrocrania, splitting of the sutures, skull demineralization and erosion or enlargement of the sella turcica. The appearance is more frequent in children with complex, rather than simple, craniosynostosis.^3^

These markings are mostly seen in the inner surface of clavarium which is marked by depressions corresponding to gyri of brain and thicker intervening bony ridges corres­ponding to cerebral sulci. The prominence of these features is dependent on constituent and age of the patient.^4^

Skull radiographs in children consistently demonstrate inner table convolutional marking and cerebral ridges which is most adequately evaluated for surgical planning with CT and 3D reconstructions.^4^ This has an advantage of currently demonstrating the intracranial complications. The associated interpretation of plain-flm skull radiography is an incidental contribution. In this patient, dental malocclusion was the complaint but, on general evaluation, beaten silver skull came out to be an incidental finding.

## CONCLUSION

The presence of beaten skull appearance may signify a disturbance in normal brain development. They may normally disappear around puberty and should not be interpreted as abnormal unless definite signs of raised intra-cranial tension are present, such as suture spreading or serial changes are present, which include macrognathia, splitting of the sutures, skull demineralization and erosion or enlargement of the sella turcica. Thus, this is a classical case where no such pathologies are associated with beaten skull appearance or it could be a varied feature of some syndrome.
